# A Case of Vitamin K Deficiency Bleeding in a Newborn: Catastrophic Yet Preventable

**DOI:** 10.7759/cureus.64098

**Published:** 2024-07-08

**Authors:** Yeka W Nmadu, Joseph Bernhard, Amanda Klawinski, Darren Klawinski, Chetan Shah, Thomas Nakagawa

**Affiliations:** 1 Pediatrics, University of Florida College of Medicine, Jacksonville, USA; 2 Emergency Medicine, University of Florida College of Medicine, Jacksonville, USA; 3 Obstetrics and Gynecology, University of South Florida, Tampa, USA; 4 Pediatric Hematology/Oncology, Nemours Children's Health System, Jacksonville, USA; 5 Pediatric Radiology, Nemours Children's Health System, Jacksonville, USA; 6 Pediatric Critical Care Medicine, University of Florida College of Medicine, Jacksonville, USA

**Keywords:** seizures, hemorrhagic disease of the newborn, newborn, bleeding, vitamin k deficiency

## Abstract

A four-week-old full-term male infant presented to the emergency department with blood in the diaper, increasing lethargy, and vomiting and was found to have multiple intracranial hemorrhages on CT. He was delivered at home and did not receive vitamin K. Coagulation studies were abnormal, and des-gamma carboxyprothrombin (DCP) was 481, diagnostic of vitamin K deficiency. He received vitamin K and required multiple antiepileptic medications for seizure control. Vitamin K deficiency bleeding (VKDB) is a preventable disease that can have devastating consequences and could present as early, classical, or late-onset. The typical presentation manifests with cutaneous, gastrointestinal, or intracranial hemorrhage most commonly in fully breastfed infants. Vitamin K prophylaxis has proven to be effective. With increasing out-of-hospital delivery and online misinformation, there is a declining administration of intramuscular vitamin K at birth. It is the responsibility of healthcare providers to properly inform patients and their families of the importance of vitamin K prophylaxis at or before the time of delivery.

## Introduction

Vitamin K deficiency bleeding (VKDB) also known as hemorrhagic disease of the newborn (HDN) can cause significant life-threatening bleeding. Presentation is classified as early, classical, or late-onset. Early onset presents in the first 24 hours of life, classical between one and seven days, and late-onset seven days to six months, but most commonly occurs between the day of life 14 and three months. The typical presentation manifests with cutaneous, gastrointestinal, or intracranial hemorrhage most commonly in fully breastfed infants [1]. The American Academy of Pediatrics recommends parenteral vitamin K administration as the most effective way to decrease the risk of VKDB and severe bleeding in newborns and young infants [2]. We report a case of a four-week-old with multiple intracranial hemorrhages and seizures due to VKDB. 

## Case presentation

A 32-year-old G2P2 (gravida 2, para 2) presented to the emergency department with her four-week-old full-term male infant because of blood noted with stool in his diaper. The birth was an uncomplicated vaginal delivery at home. After delivery, the infant did not receive vitamin K or any immunizations. The infant was exclusively breastfed and had multiple bowel movements daily. The infant had increasing lethargy and vomiting on the day of admission. The mother reported no history of prior illness and there was no history of any bleeding problems with this infant, the mother, or other family members.

**Figure 1:**
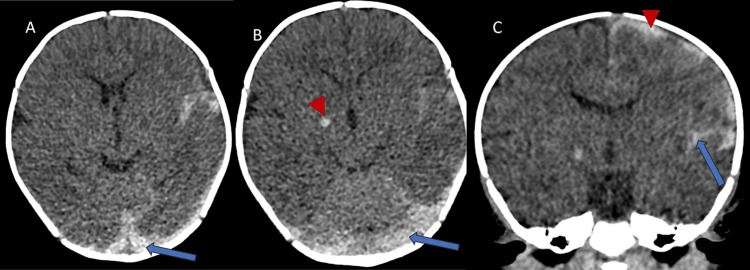
Unenhanced CT scan of head: axial images show bilateral subdural hemorrhages (arrow in A and B), right basal ganglia hemorrhage (arrowhead in B), subarachnoid hemorrhage (arrow in C) and subpial hemorrhage (arrowhead in C). There is diffuse cerebral edema with loss of gray-white differentiation

Initial examination findings were unremarkable. The patient had a blood pressure of 98/60 mm Hg, a pulse of 198 beats per minute, a respiratory rate of 37 breaths per minute, a temperature of 36.9°C (98.4°F), and a SpO2 of 97% on ambient air. The infant was irritable. No jaundice, skin bruising, contusion, or swelling of the joints was noted. The infant was not circumcised. During intravenous line placement, rhythmic movement of the right lower extremity occurred. An unenhanced CT scan (Figure 1) showed left-sided subarachnoid, left-sided subpial hemorrhages, a focal hemorrhage within the right basal ganglia, and bilateral subdural hemorrhages. There was diffuse cerebral edema with loss of gray-white differentiation.

**Figure 2:**
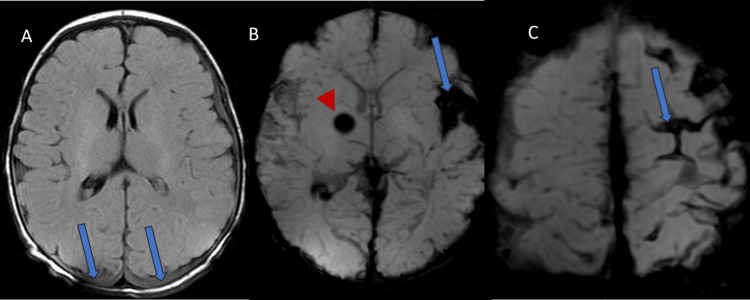
Unenhanced MRI of the brain: FLAIR axial image (A) shows bilateral subdural hemorrhages (arrows). Axial SWI, show right basal ganglia hemorrhage (arrowhead in B), subarachnoid hemorrhage (arrow in B), and subpial hemorrhage (arrow in C) FLAIR: fluid attenuation inversion recovery; SWI: susceptibility-weighted images

The infant received lorazepam and a loading dose of levetiracetam followed by maintenance dosing. Urinalysis showed a large amount of blood with greater than 182 red blood cells/high power field. Coagulation studies showed a prothrombin time (PT) of >200 seconds (normal range: 11.8-15.0 seconds), an international normalized ratio (INR) of 28.30 (normal range: </=4.00), and an activated partial thromboplastin time (aPTT) of >250 seconds (normal range: 22.9-37.8 seconds). He was admitted to the pediatric intensive care unit (PICU) for ongoing neurologic monitoring and seizure control. The infant was evaluated by neurology, neurosurgery, and hematology. Continuous electroencephalography (EEG) monitoring was instituted. The infant had ongoing clinical and electrographic seizures requiring administration of fosphenytoin and eventually, lacosamide to control his seizures. The infant did not require noninvasive or invasive respiratory support. He remained hemodynamically stable during his PICU admission. Coagulation abnormalities corrected following administration of vitamin K and remained normal in follow-up studies. The des-gamma carboxyprothrombin (DCP) was 481 ng/mL (normal range: 0.0-7.5). Follow-up unenhanced magnetic resonance imaging (MRI) again displayed subarachnoid hemorrhage in the left Sylvian fissure and subpial hemorrhages along the surface of the left hemisphere (Figure 2). A focal hemorrhage within the right basal ganglia and bilateral subdural hemorrhages along the posterior convexities and tentorium were also present. There was no evidence for deep venous sinus thrombosis.

Seizures were eventually controlled, and the infant was able to take nutrition by mouth. He was discharged home on levetiracetam and lacosamide. 

## Discussion

We report a case of VKDB that resulted in significant morbidity with intracranial bleeding and seizures. This case demonstrates the severity of this disease and how a simple decision not to give vitamin K prophylaxis to the newborn can have devastating consequences. Vitamin K is a fat-soluble vitamin necessary for the synthesis of several clotting factors such as factors II, VII, IX, and X. The newborn is deficient in vitamin K due to poor placental transfer of vitamin K, lack of vitamin K in breast milk, and poor intestinal absorption due to the relative lack of intestinal gut flora [3]. The newborn is prone to adverse bleeding events with clinical consequences ranging from very mild prolonged bleeding following circumcision to devastating intracranial hemorrhage leading to long-term disability or even death. VKDB also known as HDN is classified based upon time of presentation. Early VKDB occurs less than 24 hours post-partum, classic VKDB occurs between one and seven days post-partum, and late VKDB occurs between two weeks and six months of age [3]. Our patient developed classical VKDB. Late-onset VKDB is typically the most severe presentation resulting in 60% of infants presenting with an intracranial hemorrhage and a mortality rate of 14% [4]. Infants who do not receive vitamin K prophylaxis in the peri-partum period have increased odds (81) of developing late-onset VKDB [5].

Diagnostic criteria for VKDB include a PT greater than four times the upper limit of normal and one of the following: (1) normal/increased platelet count with normal fibrinogen and absence of fibrin degradation products, (2) return PT level to normal limits within 30 minutes of administration of intravenous vitamin K, and (3) elevation in levels of proteins induced in vitamin K absence (PIVKA-II) or DCP, an inactive precursor of prothrombin elevated in the absence of vitamin K [6]. Our patient fulfilled all three criteria. The World Health Organization currently recommends neonates receive a single intramuscular dose of vitamin K (0.5-1 mg) at birth [7]. Oral formulations of vitamin K are available; however, they have not been proven to be as efficacious as parenteral administration [8]. The data surrounding vitamin K prophylaxis in the prevention of VKDB is compelling. Vitamin K prophylaxis markedly reduces the risk of both severe VKDB (relative risk: 0.19 (0.08-0.46)) and bleeding at circumcision (relative risk: 0.18 (0.08-0.42)) [9]. In order to prevent VKDB following circumcision as little as nine infants need to be treated with vitamin K and to prevent moderate to severe VKDB the number needed to treat is 74 [9]. The severity of VKDB is exemplified in our case. The strength of the data supports vitamin K prophylaxis for all neonates given the relative ease of administration and cost-effectiveness.

A multitude of reasons exist why parents refuse vitamin K prophylaxis for their child, despite the devastation that can ensue if not administered. There has been a 77% increase in out-of-hospital births, increasing the birth rate to 1.61% for all out-of-hospital births in the United States from 2004 to 2017 [10]. From 2011 to 2012, out-of-hospital births increased from 1.26% to 1.36% [11]. In 2017, one out of 62 births were out-of-hospital births [10]. This rate will likely continue to increase as additional legislation is passed, allowing more autonomy for midwives, and giving parents more options for their birth experience. The refusal of intramuscular vitamin K administration for out-of-hospital home births is 14.5% and 31% in non-hospital birthing centers [11]. Based on these statistics from 2011 to 2012, approximately 12000 to 25000 newborns born out-of-hospital are at risk for HDN every year [11]. Canadian investigators demonstrated an association between refusal of vitamin K administration in homebirths as well as midwife-assisted deliveries [12]. Investigators in Michigan found similar situations where many parents were not properly informed of the consequences of their newborn not receiving vitamin K with out-of-hospital births [13]. In a cross-sectional survey of mothers in the United States, factors most commonly associated with refusal of intramuscular vitamin K administration included non-Hispanic Caucasians, >30 years of age, college graduates, and/or breastfeeding [14]. Other major contributing factors to parental refusal of intramuscular vitamin K administration include (1) creating a “natural” birth experience, (2) fear of harm from preservatives in vitamin K, (3) concerns that vitamin K injection will be painful for the infant, and (4) mistrust of medical professionals [11,15]. Parental refusal for newborn vitamin K prophylaxis was also associated with other preventative healthcare services such as immunizations [11]. Birth trauma also affects how women and their partners view healthcare professionals and the healthcare system. A desire for a more natural or even gentle birth experience can be influenced by internet and social media information about traumatic birthing experiences creating distrust and altering decision-making regarding recommendations for themselves and their newborns. Frederick Leboyer describes the method of “gentle birth” as designed to decrease stimuli and decrease trauma for the infant during birth [16]. The method includes decreased lighting, a quiet delivery room, delayed cord clamping, gently stoking the infant after being placed on the maternal abdomen at delivery, and a warm bath in the room [16]. This method did not mention avoiding vitamin K administration. Vitamin K is routinely administered within an hour or so after delivery depending on policies and the attending provider if out-of-hospital birth is performed. A birth that is non-traumatic or less traumatic to the infant does not negate the need or recommendation for vitamin K administration, which is sometimes a misconception.

The refusal of preventative healthcare measures such as vitamin K prophylaxis has been fueled by internet use to gather medical recommendations [15]. Many search engines generate articles and videos with misleading or false information regarding medical made for their children. Parental knowledge about specific interventions such as vitamin K prophylaxis should be assessed so healthcare professionals may better present necessary and accurate information regarding potentially life-saving interventions. A survey of parental refusal of vitamin K prophylaxis for their children noted that most respondents could not state any contraindications to prophylaxis, complications of not receiving vitamin K, or the sequelae of HDN [15,19]. Healthcare interventions require that patients and families be informed of treatment risks and benefits. Prior to delivery, mothers-to-be and their partners should be provided information about VKDN and the importance of vitamin K injections for newborns. An online printable resource addressing frequent questions and myths provided through evidence-based birth is available to midwives, other providers, and the general public [20]. This resource references several studies and specifically mentions the risk associated with failure to administer vitamin K [20]. Parental concerns should be acknowledged in a non-judgmental manner while providing accurate information in an objective way that is understandable to patients and families. Parental decisions regarding vitamin K administration and other preventative measures are typically made well before delivery, indicating several opportunities for healthcare providers to bridge these discussions [15,19].

## Conclusions

VKBD is a preventable disease. Devastating consequences including life-threatening intracranial hemorrhage and death can occur if vitamin K is not administered at birth. Healthcare professionals should be well informed of the adverse consequences of VKDB that can be life-threatening so parents can be properly informed of the importance of vitamin K prophylaxis. Resources should be provided to the mother or mother to be and partner allowing them to review the benefits and risks and further empowering their informed decision making. These discussions should occur prior to and at the time of delivery, especially in cases where an out-of-hospital birth occurs.
